# Compact resonant systems for perfect and broadband sound absorption in wide waveguides in transmission problems

**DOI:** 10.1038/s41598-022-13944-1

**Published:** 2022-06-15

**Authors:** Jean Boulvert, Gwénaël Gabard, Vicente Romero-García, Jean-Philippe Groby

**Affiliations:** Laboratoire d’Acoustique de l’Université du Mans (LAUM), UMR 6613, Institut d’Acoustique - Graduate School (IA-GS), CNRS, Le Mans Université, Le Mans, France

**Keywords:** Acoustics, Applied mathematics

## Abstract

This work deals with wave absorption in reciprocal asymmetric scattering problem by addressing the acoustic problem of compact absorbers for perfect unidirectional absorption, flush mounted to the walls of wide ducts. These absorbers are composed of several side-by-side resonators that are usually of different geometry and thus detuned to yield an asymmetric acoustic response. A simple lumped-element model analysis is performed to link the dependence of the optimal resonators surface impedance, resonance frequency, and losses to the duct cross-sectional area and resonator spacing. This analysis unifies those of several specific configurations into a unique problem. In addition, the impact of the potential evanescent coupling between the resonators, which is usually neglected, is carefully studied. This coupling can have a strong impact especially on the behavior of compact absorbers lining wide ducts. To reduce the evanescent coupling, the resonators should be relatively small and therefore their resonances should be damped, and not arranged by order of increasing or decreasing resonant frequency. Finally, such an absorber is designed and optimized for perfect unidirectional absorption to prove the relevance of the analysis. The absorber is 30 cm long and 5 cm thick and covers a single side of a 14.8 × 15 cm^2^ rectangular duct. A mean absorption coefficient of 99% is obtained experimentally between 700 and 800 Hz.

## Introduction

Wave absorption in reciprocal scattering problems with compact absorbers has been actively studied during the past years due to the possibilities ranging from fundamental Physics to applications in several branches of science and technology. In one-dimensional reciprocal scattering problems, when an obstacle embedded in a surrounding material is radiated by an incident wave, the incident energy is reflected, transmitted, or absorbed. These scattering properties strongly depend on the geometry and size of the obstacle as well as on the frequency-dependent properties of both the obstacle and the surrounding medium. While the perfect absorption of waves requires the suppression of both the reflected and transmitted energies at the same frequency (critical coupling condition), the compactness of the absorbers requires the use of locally resonant materials or metamaterials placing the working frequencies in the deep subwavelength regime. In this 1D scattering problem, two main configurations can be considered. The first one solely consists in the reflection problem, i.e., there is no transmitted waves in the complete range of frequencies. In this case, perfect absorbers have been developed for acoustic^1^, elastic, and electromagnetic^2^ waves. The second configuration consists in the full transmission problem. In this case, two different situations can be analyzed if the scattering is symmetric or antisymmetric. When the obstacle is mirror-symmetric, its reflection and transmission coefficients, *R* and *T* respectively, do not depend on the incidence side. Then, perfect absorption, $\alpha = 1-|R|^2-|T|^2=1$, can only be obtained by using degenerate resonators, i.e., resonators presenting monopolar and dipolar resonances at the same frequency^3–6^. If the resonators are monopolar or dipolar, only quasi-perfect absorption can be achieved^7^. When the obstacle is not mirror symmetric, the scattering depends on the incidence side, meaning that the reflection coefficients, $R^+$ and $R^-$, are different for each side. Then, unidirectional perfect absorption, $\alpha ^+ = 1-|R^+|^2-|T|^2=1$, can be obtained at a single frequency^8^. A cascading design procedure can then be used to place several perfect absorption peaks with low quality factors in order to produce broadband unidirectional perfect absorption^9,10^.

Inspired by the previous results, perfect sound absorbers have been engineered during the last years for different situations. Panels made of Helmholtz Resonators (HRs) or membranes have been used to design perfect absorbers for the reflection problem^11,12^ and in the full scattering problem^13^. Degenerate resonators have been developed by using HRs and membranes for the perfect absorption for the full scattering problem. Finally, flush mounted asymmetric absorbers (FMAAs) in acoustic ducts have been designed and studied in the literature for the unidirectional perfect absorption in reciprocal and asymmetric scattering problems. Various case studies have been reported in the literature: the duct is straight^8,10,14–18^ or of varying cross section^9^, the resonators are placed far apart^8,10,14–16^ or side-by-side^9,17,18^, the resonators are identical and assembled in groups^15^ or are tuned but have different losses^10^ or are detuned^8,9,14–18^, the resonators are assembled in parallel, i.e., different resonators are located at the same position^10,14,15^, or in cascade^8–10,14,16,17^. However, all these cases are focused on two main configurations: (i) wide ducts with spaced resonators, allowing perfect absorption at the condition $k_0 \delta \approx \pi$ (where $k_0$ is the wave number and $\delta$ is the distance between resonators), so for large distance between the scatterers and as a consequence for non-compact systems^10^, or (ii) side-by-side resonators with narrow ducts, allowing the suppression of the transmission through a large range of frequencies in which the reflection suppression is produced by the critical coupling condition^9^. Therefore, no study faces the important case of compact systems composed of side-by-side resonators and mounted in a waveguide of relatively large and constant cross section^19^ for perfect absorption. In this configuration, perfect absorbers fully cancel sound waves while allowing air and light to pass through. This way, neither geometrical variations of the ducts that can damage their ventilation capacity, nor the radiation of the ducts are taken advantage of and the acoustic behavior of the absorbers only arises from their resonances.

In this work, we pay special attention to asymmetric absorbers flush mounted to the walls of ducts of constant and large cross section. The simplest absorber is composed of a downstream resonator that act as a soft wall around its resonance frequency and of an upstream resonator that impedance matches the system at the same frequency. On the one hand, expressing the conditions for the resonators to be optimal in terms of surface impedance highlights the strong impact of the cross sectional area of the duct and of the resonator spacing. The combination of more than two resonators in parallel or in cascade can be used to obtain multiple soft walls effective around different frequencies and/or to obtain an impedance match around multiple frequencies. On the other hand, the potential evanescent coupling, i.e., the interaction due to evanescent waves, between the resonators can also have very strong and undesired effects on the absorption properties of the system, especially in three different configurations: (i) when the resonators are close to each other, (ii) when the duct is wide, and (iii) when the resonators are weakly damped. We provide simple explanations of the underlying physical principles considering the effects of both the evanescent coupling between resonators and the large duct cross section on the absorption capabilities of the system. The results are general and can be extended to asymmetric absorbers mounted inside the ducts or to ducts of variable cross section.

In this work, and for concision, the term “resonator” qualifies a single degree of freedom (1-dof) resonator, i.e., a resonator having a single resonance that is controlled by the tuning of its geometric parameters.

## Results

### Asymmetric absorber with side-by-side flush mounted resonators

**Figure 1:**
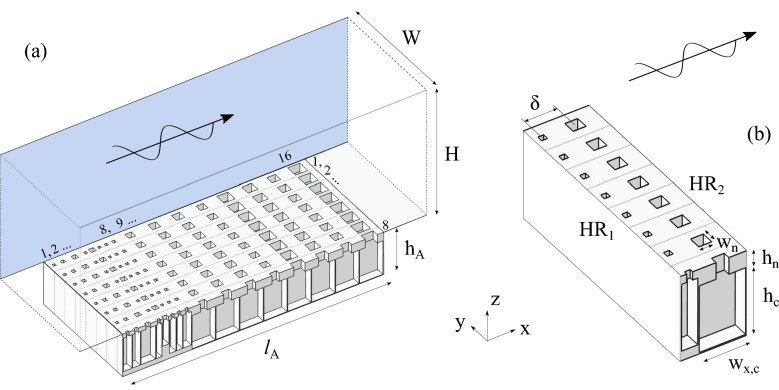
Optimized compact and broadband FMAA for $W \times H= 14.8 \times 15$ cm$^2$ duct. The incident sound wave propagates in the positive *x* direction. (**a**) Sectional view. (**b**) Detail of the sectional view showing two columns of HRs.

Figure 1a shows an example of configuration analyzed in this work. Acoustic waves propagate in a straight waveguide of rectangular cross sectional area and loaded on a single face by side-by-side HRs forming a FMAA. In the illustration, the system is made of 16 resonator columns along the *y* direction with 8 HRs on each of them. The resonators are HRs formed of two rectangular cross sectional waveguides playing the role of neck and cavity as shown in the details of Fig. 1b [subindex *n* (*c*) refers to neck (cavity)]. The columns in the system are made by identical resonators fully covering the bottom side of the main waveguide, while resonators are different between rows. In this way, the system is asymmetric $(R^+\ne R^-)$ and reciprocal $(T^+= T^- \equiv T)$.

The depicted FMAA is optimized for $W \times H = 14.8 \times 15$ cm$^2$ cross sectional waveguides to achieve the maximal absorption capabilities over a target frequency range by using the metaheuristic Particle Swarm Optimization (PSO) algorithm (see Methods-Optimization for more details).

### Generic explanation of the efficiency of flush mounted asymmetric absorbers

We first analyze what are the effects of the duct cross section and of the resonator spacing on the monochromatic and broadband absorption properties of this kind of absorbers by using simple modeling and arguments.

To explain the operating principles of1 FMAAs, the simplest FMAA presented in Fig. 1b is considered. It is composed of a pair of HRs axially spaced by a distance $\delta$. Each resonator is replicated in the lateral direction of the duct, *y*, forming two resonator columns composed of *N* resonators. The duct is straight, of constant cross sectional area $W\times H$, and is assumed to be infinitely long. The FMAA is optimized to obtain perfect upstream absorption at a given frequency $f_0$ smaller than the first cut-off frequency of the waveguide. The downstream resonators (HR$_2$) stops the propagation of the sound wave by reflecting it back while the upstream resonators (HR$_1$) cancels the reflected wave and thus allows the pair of resonator columns to cause no reflection in the upstream direction^8,9^.

To analyze the effect of the geometry on the perfect absorption properties, a one-dimensional analysis is performed by the Transfer Matrix Method (TMM). Point resonators and plane wave propagation are assumed, so no evanescent coupling is considered, see Methods-TMM. Using an implicit time dependence $\text {e}^{\text {i}\omega t}$, the impedance conditions to obtain perfect upstream absorption are as follows:

1 $$\begin{aligned}&{T}(f_0)=0,~&Z_2(f_0) = 0,&\qquad\qquad~\text {if HR}_{2}\text { is lossless}, \end{aligned}$$

2 $$\begin{aligned}&{T}(f_0)\approx 0,~&\frac{Z_2(f_0)}{ S_2} \ll \frac{N}{S_d},&\qquad\qquad~\text {if HR}_{2}\text { is lossy}, \end{aligned}$$

3 $$\begin{aligned}&{R}^+(f_0) = 0,~&\qquad\qquad\frac{Z_1(f_0)}{ S_1} = \frac{N}{S_d} \left( \sin ^2(k_0 \delta )-\text {i}\frac{\sin (2k_0 \delta )}{2}\right) ,&\qquad\qquad~\text {with}~ k_0 \delta \ne 0 ~\text {mod}~ \pi , \end{aligned}$$

with $Z_{1,2}$ the surface impedance of HR$_{1,2}$, $S_{1,2}$ the area of HR$_{1,2}$ connected to the duct, $S_d=H W$ the cross sectional area of the duct and $k_0$ the duct wave number, see Fig. 1b. As the resonators are HRs of square cross section necks, $S_1 = w_{n,1}^2$ and $S_2 = w_{n,2}^2$.

Let us first analyze the no-transmission condition at $f_0$ by only using the resonators HR$_2$. The reactance, i.e., the imaginary part of the impedance of a resonator, is null at its resonance frequency while its resistance, i.e., the real part of the impedance, can only be null if the resonator is lossless. This way, if HR$_{2}$ resonates at $f_0$ and is lossless, it generates a perfect soft wall at $f_0$ no matter the number of resonator *N* along the y-axis, see Eq. (1), and the incident wave is fully reflected. In practice, the losses of a resonator are never null due to viscous and thermal dissipations. Thus, the soft wall can only be quasi-perfect. To strongly reduce the transmission around $f_0$, resonators HR$_{2}$ must resonate and have a low resistance at $f_0$, see Eq. (2). Then, $|{T}(f_0)|$ is proportional to the ratio $Z_2(f_0) S_d/(N S_2)$.

**Figure 2:**
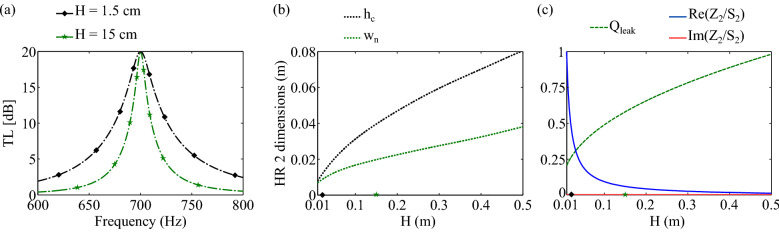
Overview of the TMM prediction of the HR$_2$ behavior mounted in a rectangular section duct and optimized for TL (700 Hz) = 20 dB. (**a**) TL(*f*) for $H=1.5$ cm and for $H=15$ cm. (**b**) Optimized HR$_2$ cavity height, $h_c$, and neck width, $w_n$. (**c**) Optimized HR$_2$ behavior at 700 Hz in terms of normalized leakage quality factor, $Q_{leak}$, and in terms of normalized surface impedance, $Z_2/S_2$. The diamond and star markers on the abscissa axis of (**b**) and (**c**) indicate that *H* is equal to 1.5 cm and 15 cm, respectively.

To better understand the impact of the duct cross-sectional area, $S_d$, on the attenuation, a parametric study is performed. Only HR$_2$ is considered and optimized for $H \in [1; 50]~$cm, such that at $f_0=700$ Hz, $|T|=0.1$, i.e., such that the Transmission Loss (TL) is TL $=-20\log (|{T}|)=20$ dB, see Fig. 2a. The optimized dimensions of HR$_2$ are $h_c$ and $w_n$ while the other dimensions of the problem are fixed: $W=w_{c,x}=w_{c,y}=5~$cm and $N=1$. Note that *N* and $S_d$ have inversely proportional effects, see Eq. (2). Thus, the most meaningful variable is $S_d/N$.

The minimum of transmission appears at the HR$_{2}$ resonance frequency $f_0$ with a null reactance and a resistance which decreases with increasing *H* as shown in Fig. 2c. Actually, as $f_0$ is fixed as *H* increases, the optimal $h_c$ and $w_n$ increase to lower the resistance, see Fig. 2b. This translates in the fact that the width of the TL peak decreases, i.e., the quality factor of the resonator mounted in the duct, $Q_{leak}$, see Methods—Computation of $Q_{leak}$, increases, see Fig. 2c. It means that the peak of the minimal transmission will be narrower as *H* increases.

Considering that the incident wave is reflected back by a soft wall formed by the HR$_2$ column, an impedance match at the HR$_1$ column position and at $f_0$ is obtained if the HR$_1$ impedance is that given by Eq. (3). For the dimensionless distance $k_0 \delta =0 \mod \pi$, i.e., when the two resonator columns are at the same position or when they are separated by a distance $\delta =\lambda /2 \mod \lambda$, the Fabry–Perot interferences of the absorber forbid the impedance match to happen; the absorber is then fully reflective^8^. Apart from these specific spacings and according to the TMM analysis, the impedance match can be perfect because the optimal resistance is not null. The optimal impedance of HR$_1$ thus depends on two variables: $S_d/N$ and $k_0 \delta$.

**Figure 3:**
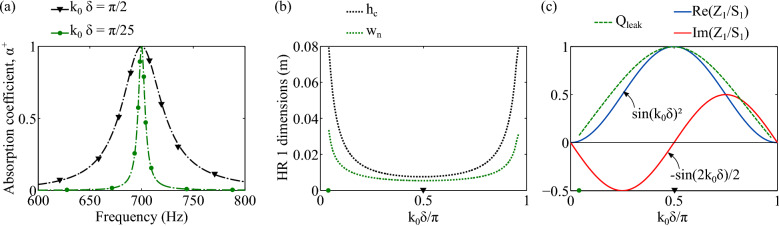
Overview of the TMM prediction of a FMAA behavior mounted in a rectangular section duct of height $H=15$ cm and such that HR$_1$ is optimized for $\alpha ^+(700~{\mathrm {Hz}})=1$ with $k_0(700~{\mathrm {Hz}})\delta \in ~]0; \pi [$ and with a perfect soft wall at a distance $\delta$. (**a**) $\alpha ^+(f)$ of optimized FMAA for $k_0(700 {\mathrm {Hz}})\delta$ $= \pi /2$ and for $k_0(700 {\mathrm {Hz}})\delta$ $= \pi /25$. (**b**) Optimized HR$_1$ cavity height, $h_c$, and neck width, $w_n$. (**c**) Optimized HR$_1$ behavior in terms of normalized leakage quality factor, $Q_{leak}$, and in terms of normalized surface impedance, $Z_1/S_1$. The dot and triangle maker on the abscissa axis of (**b**) and (**c**) indicate that $k_0(700~{\mathrm {Hz}})\delta$ is equal to $\pi /25$ and $\pi /2$, respectively.

To better understand the impact of $k_0 \delta$, another parametric study is performed with $W=w_{c,x}=w_{c,y}=5~$cm, $H=15$ cm, and $N=1$. A perfect soft wall is placed at a downstream distance $\delta$ of the HR$_1$, which is the only HR considered and optimized such that at $f_0=700$ Hz, $\alpha ^+=1$, with $k_0(f_0)\delta \in ]0 ; \pi [$, see Fig. 3a. Because of the trigonometric functions in the expression of the optimal impedance of HR$_1$, the impact of $k_0 \delta$ is $\pi$-periodic. For $k_0 \delta$ close to $0 \mod \pi$ (resp. $\pi /2 \mod \pi$), the optimal resistance is minimal (resp. maximal); the HR$_1$ must present small (resp. high) intrinsic losses, i.e., low (resp. high) $Q_{leak}$, see Fig. 3c. This is obtained with a wide (resp. small) neck and a large (resp. small) cavity, see Fig. 3b. As a consequence, the absorption peak is narrow (resp. large). The optimal reactance is lower (resp. larger) than zero for $k_0 \delta \in ]0; \pi /2[ \mod \pi$ (resp. $k_0 \delta \in ]\pi /2; \pi [ \mod \pi$) which implies that the resonance frequency of HR$_1$ is higher (resp. lower) than $f_0$. HR$_1$ is then slightly detuned with HR$_2$^8,9,14^ except for $k_0 \delta = \pi /2 \mod \pi$ where both resonators are tuned at the same frequency, $f_0$^10,15^.

The impacts of $S_d/N$ to reach the impedance match are similar to its impacts on the attenuation. In particular, the larger $S_d/N$, the larger the optimal $h_c$ and $w_n$ of HR$_1$ to obtain a smaller surface resistance. The width of the absorption peak obtained with an optimal HR$_1$ reduces as $S_d/N$ increases and *vice versa*. This way, the optimal HR$_1$ is usually more lossy than HR$_2$^8,10,14^ because HR$_2$ should have a very low intrinsic losses to effectively generate a soft wall. However, if the attenuation generated by HR$_2$ is moderate and if $k_0 \delta$ is close to 0, then HR$_1$ can be less lossy than HR$_2$.

Finally, a FMAA for perfect absorption can be composed of a unique replicated resonator. To do so, the distance between the resonators columns should be such that $k_0 \delta = \pi /2 \mod \pi$ because all the resonators forming the absorber have the same resonance frequency. In addition, the number *N*^15^ or $S_d$ should be different at the HR$_1$ column position than at the HR$_2$ column position to satisfy both Eqs. (2) and (3) with $Z_1/S_1=Z_2/S_2$.

Now that the monochromatic perfect absorption has been discussed, we move to the problem of broadband perfect absorption.

A broad low-transmission frequency range can be obtained with a single resonator if the duct is very narrow, see Eq. (2). A high and large TL peak is formed around the resonance frequency of the resonator resulting in a no-transmission frequency region^9^. To obtain an impedance match at *F* targeted frequencies spanning the broad frequency range of interest with a single upstream resonator, its surface impedance should be equal to the optimal impedance, Eq. (3), at the *F* targeted frequencies. This is not possible because the impedance of 1-dof resonators have natural frequency variations that do not follow that of the optimal impedance. Then, only a monochromatic peak of perfect absorption can be formed and a small section duct and a resonator spacing such that $k_0 \delta = \pi /2 \mod \pi$ all broaden the peak without forming a plateau. This is the reason why it is important to analyze the transmission of the absorbers in a logarithm scale, i.e., the TL, and the absorption in a linear scale, i.e., $\alpha$.

This way, only monochromatic peaks of absorption can be obtained with a single pair of resonators. Then, we tackle the problem of broadband perfect absorption by using more than two resonators. We notice here that perfect absorption can only be obtained at discrete frequencies and not over a broadband range of frequencies. The broadband perfect absorption discussed in this work is achieved by a combination of discrete perfect absorption frequencies with very low quality factor peaks, thus allowing a broadband absorption close to one between the perfect absorption peaks^20,21^. Typical cases to target perfect absorption at *F* frequencies are discussed as follows and summarized in Table 1.

As mentioned before, in narrow ducts, a single resonator can induce a low-transmission over a broad frequency range. If *F* upstream resonators are used, each one of them impedance matches the system at one of the *F* targeted frequencies. A total of $1+F$ resonators form the system^9^.

In wider ducts, resonators of reasonable volume only cancel the transmission around their resonance frequency. Thus, *F* resonators tuned at the *F* targeted frequencies are required to obtain *F* attenuation peaks that can result in a strong attenuation over a broad frequency bandwidth^10,16,22^. The resonators canceling the transmission can be assembled in parallel, i.e., located at the same duct axial position^10^. These resonators generate an equivalent resonator whose admittance is equal to the sum of the resonators admittances. The equivalent resonator reflects back the impinging wave around the resonance frequencies of its composing resonators. Then, a single upstream resonator is not enough to obtain *F* impedance match frequencies. Conversely, *F* upstream resonators are enough and a total of 2*F* resonators is used. The resonators canceling the transmission can also be assembled in cascade, i.e., not located at the same duct axial position^16^, and each resonator in turn reflects back the impinging wave around its resonance frequency. In this case, a single upstream resonator can be used to obtain an impedance match at *F* frequencies. To do so, the ordering of the resonators and their spacing must be optimized. In fact, the impedance of the upstream resonator must follow Eq. (3) with $\delta$ being different for each target frequency. A total of $F+1$ resonators is then used^16^.

**Table 1:** Broadband perfect absorption, number of resonators.

Broadband soft wall	Broadband impedance match	
1 Resonator, Small section duct	1 Resonator: No Unreachable optimal impedance	F Resonators: Yes Each resonator is responsible for one frequency
F Resonators In parallel	1 Resonator: No Unreachable optimal impedance	F Resonators: Yes Each resonator is responsible for one frequency
F Resonators In cascade	1 Resonator: Yes $\delta$ is different for every pair of resonators	F Resonators: Yes Each resonator is responsible for one frequency

This analysis only considers the effects of the resonators around their target frequency. However, all the resonators impact the reflection and the transmission of the whole system at all the frequencies. This impact can be strong. For instance, a resonator intended to impedance match the system at $f_0$ such that $k_0 \delta \approx 0 \mod \pi$ have a small optimal resistance at $f_0$. Thus, its resonance is weakly damped which induces a non negligible attenuation/reflection around its resonance frequency. Similarly, a resonator intended to cancel the transmission at $f_0$ can also reduce the reflection of the system at another frequency by forming an unintended coupling with another resonator^7^. On the one hand, these unintended attenuations can be exploited to reduce the number of resonators dedicated to the attenuation but on the other hand, these unintended reflections may require additional resonators to preserve the impedance match at the target frequencies. The accumulation of these second order effects can strongly impact the behavior of the whole absorber and lead to very specific configurations where the number of resonators can be lower than $F+1$.

### Effects of evanescent coupling on the perfect absorption conditions of asymmetric absorbers

The one dimensional analysis highlights the main mechanisms of the FMAAs but relies on simplifying hypothesis. In particular, the potential evanescent coupling between the resonators is neglected. Its effect on the FMAAs behavior has never been discussed before. Evanescent coupling was only partially investigated to enhance the TL in^23^ to our knowledge. A first step towards general predictions of the behavior of flush mounted resonant systems consists in using a Mode Matching Technique (MMT)^17^. The MMT accounts for the resonators by their surface impedance and high-order modes are considered in the main duct which allows to consider the potential evanescent couplings between resonators. In this work and for computational efficiency, the modal decomposition is performed using the Chebyshev polynomials, see Methods-MMT.

**Figure 4:**
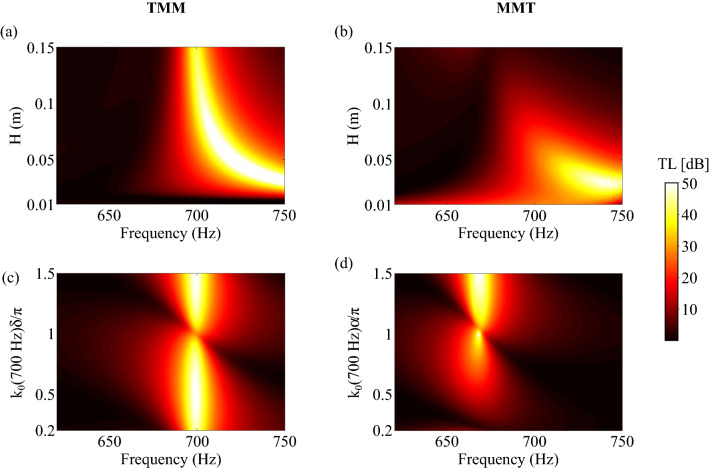
TL predictions of a FMAA composed of two identical and axially spaced HRs. The predictions are either performed by the TMM (**a**,**c**) or by the MMT (**b**,**d**). In (**a**,**b**), the distance between the HRs is $\delta =5$ cm and the height of the duct, *H*, varies between 1  and 15 cm. In (**c**, **d**), $\delta \in [5; 37]$ cm, i.e., $k_0(700 {\mathrm {Hz}})\delta \in [0.2; 1.5]\pi$, and $H=15$ cm.

A case study is performed to illustrate the impacts of the evanescent coupling. The HR leading to a TL(700 Hz$$)=20$$ dB with $$H = 15$$ cm, according to the TMM, is selected and duplicated along the duct axial direction. The TL of the system is computed with the TMM and with the MMT. First, the height of the duct *H* is varied between 1  and 15 cm with a constant axial separation distance between the resonators $$\delta = 5$$ cm, i.e., $$k_0(700 {\mathrm {Hz}})\delta =0.2\pi $$, see Fig. 4a,b. Then, $$\delta $$ is varied between 5  and 37 cm, i.e., $$k_0(700 {\mathrm {Hz}})\delta \in [0.2; 1.5]\pi $$, and $$H=15$$ cm., see Fig. 4c,d.

According to the TMM, the lower the duct height, the higher the resonance frequency (corresponding to the TL pick frequency) of the HR. This is due to the length correction in the expression of the impedance of the neck accounting for the duct height impact on the HR radiation. In addition, the TMM predicts that the highest (resp. lowest) TL peak value is obtained for a spacing between the resonators such that $k_0(f_0) \delta = \pi /2 \mod \pi$ (resp. $0 \mod \pi$). The MMT is in good agreement with the TMM in terms of resonance frequency variation although a small shift is observed. The main difference between the two methods is that, according to the MMT, the TL drops for high ducts and close resonators. This reduction of the attenuation of the resonators is mainly due to the evanescent coupling between the two resonators. In addition, resonators having weakly damped resonances, i.e., small intrinsic *Q*-factors, are more affected by the effects of the evanescent coupling.

### Optimized compact flush mounted asymmetric absorber for wide duct and broad target frequency range

To validate the present analysis, a compact FMAA with length $l_A=30$ cm and height $h_A=5$ cm, for $W \times H = 14.8 \times 15$ cm$^2$ section duct has been designed, optimized, manufactured, and tested experimentally, see Methods—Experimental set-up. The FMAA covers a single face of the duct and is composed of 16 different HRs replicated 8 times along the row, see Fig. 1. The open area ratio of the system is thus $HW/(HW+h_AW)=$ 75%. The target frequency bandwidth is $f \in [700;800]$ Hz, i.e., $\lambda \in [8.6;9.8]h_A$, with $\lambda$ the wavelength in air. Then, the FMAA is sub-wavelength in height. The optimization method is based on predictions accounting for the potential coupling between the resonators and is presented in Methods - Optimization. The evanescent coupling impacts the behavior of the absorber but leaves the possibility to reach an absorption coefficient approaching 99% over the sub-wavelength and 100 Hz broad target frequency range.

**Figure 5:**
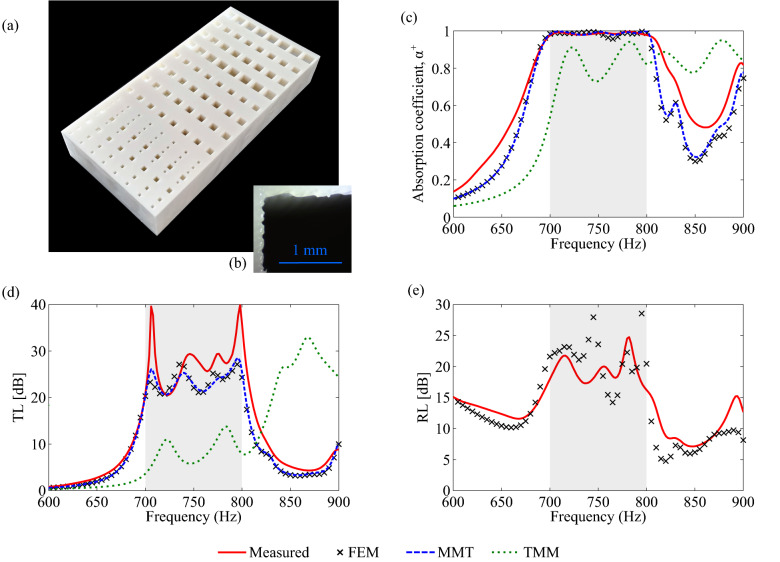
Optimized compact and broadband absorber for $H=15$ cm high duct. (**a**) Picture of the tested sample. (**b**) Microscope picture of a HR neck. (**c**) Absorption coefficient, $\alpha ^+$. (**d**) Transmission loss, TL$=-20 \log (|T|)$. (**e**) Reflection loss, RL$=-20 \log (|R^+|)$. Behavior obtained by measurements (solid lines), FEM (crosses), MMT (dashed line) and TMM (doted line).

The resulting optimized and manufactured FMAA is presented in Fig. 5a and its geometric parameters are summarized in Table 2. The 8 upstream HRs (n$^{\circ }$ 1 to 8) possess thinner neck and smaller cavity volume than the 8 downstream HRs (no 9 to 16). Then, the upstream and the downstream HRs occupy $27\%$ and $73\%$ of the FMAA volume, respectively. Its computed and measured acoustic behavior are shown in Fig. 5 in terms of (c) Absorption, $\alpha ^+$, (d) TL and (e) RL $= -20 \log (|{R}^+|)$. Experimentally, $\alpha ^+ \in [97.5; 99.5]\%$ and ${\bar{\alpha }}^+ =98.5\%$, TL $\in [20; 40]$ dB and RL $\in [17; 25]$ dB, in the 100 Hz broad target frequency region. The designed FMAA is then compact, sub-wavelength, and achieves experimentally almost $99\%$ of sound absorption over a broad target frequency range.

As expected, the TMM prediction is not in good agreement with that of the Finite Element Method (FEM) (see Methods—Finite Element Method), while the MMT prediction is, due to the impact of evanescent coupling on the behavior of the FMAA. In addition, the MMT and FEM predictions are in good agreement but small differences exist because the HRs are modeled as surface impedance in the MMT while their exact geometry is modeled by FEM. The correlation between the measurements and the FEM prediction is good and higher TL peaks values can be observed experimentally. This is due to the presence of small corrugations in the the lateral edges of the necks of the HRs, see Fig. 5b. This manufacturing defect increases the losses, the resistance, and the damping of the HRs while having a negligible impact on their resonance frequency^24^. Because of the resistance increase, the TL of each HR and of the FMAA should be reduced according to the TMM, see Eq. (2). However, the TL of the FMAA is increased in the studied case compared to numerical models not considering this extra damping and because of the presence of the evanescent coupling: all the HRs are more damped and thus the evanescent coupling that usually reduces the TL of groups of resonators is weaker resonators is weaker, implying an increase of the TL peaks. Finally, the measured RL presents smaller peaks and dips than the RL predicted by the FEM. This is an expected effect that can be observed both by the TMM and the numerical models when increasing the resistance of the HRs. It indicates that it is not linked to evanescent coupling effects but mainly to the underestimation of the resistance.

The number and size of the HRs forming the optimized FMAA are analyzed in Methods—Design of compact FMAA for wide duct. We note here that the HRs responsible for blocking the sound propagation, i.e., the HRs no 9 to 16, are not tuned in a monotonic order, see Table 3. This way, HRs tuned at close frequencies are separated of a larger distance than if they they were tuned in a monotonic order. This contributes in reducing the impact of the evanescent coupling. Also, half of the resonance frequencies of these HRs is not located in the target frequency region while these HRs have an important impact on the absorption and TL in this frequency region. This scenario is not met if the FMAA is optimized with the TMM predictive model which indicates that the evanescent coupling is the reason for this unexpected situation. Finally, the HRs responsible for the impedance match, i.e., the HRs no 1 to 8, are also not tuned in a monotonic order. This is expected because their resonance frequencies are almost never the frequencies at which they are the most efficient and depend on the distance with their corresponding soft wall resonator.

**Table 2:** Geometrical parameters of the HRs composing the optimized FMAA.

HR	1	2	3	4	5	6	7	8	9	10	11	12	13	14	15	16
$h_n$	*5.00*								*10.00*							
$h_c$	40.00				28.17	40.00			*40.00*							
$w_{n}$	3.17	3.87	5.34	3.01	5.67	2.90	3.10	3.41	7.41	8.97	9.33	8.21	12.80	11.22	9.85	13.99
$w_{x,c}$	9.83	8.80	16.75	6.42	7.37	7.37	6.70	9.37	*26.70*							
$w_{y,c}$	*17.60*															

All dimensions are in mm. The values in italic correspond to the parameters values that are fixed before the optimization.

**Table 3:** Resonance frequency in Hz of the HRs composing the optimized FMAA.

HR	1	2	3	4	5	6	7	8	9	10	11	12	13	14	15	16
$f_r$	720	890	845	840	1565	765	845	780	720	840	870	785	1095	995	905	1165

### Performance evaluation

FMAAs optimized for perfect absorption are often showcased by means of performance qualifying terms, the most common of which are “sub-wavelength”, “broadband”, and high “open area ratio” (OAR)^25^. These terms are sometimes misused or misinterpreted. It is therefore important to define them carefully in light of the above analysis of FMAAs.

At normal incidence, acoustic treatments rigidly backed and of height *h* are qualified as “sub-wavelength” if they reach perfect absorption for a wavelength in air $\lambda$ such that $\lambda \gg 4h$. This comparison comes from the analysis of the simplest type of resonators: quarter-wavelength resonators (QWRs) filled with air, that can reach perfect absorption for $\lambda \approx 4h$^20,26^. In practice, the term “sub-wavelength” is used to emphasize the compactness of a treatment. The same practice is also followed with FMAAs for perfect absorption: $\lambda$ is compared to $l_A$, the length of the considered FMAA. This is because the absorption coefficient usually describes the behavior of rigidly-backed treatments. Although $l_A$ can impact the practical use of a FMAA, it has no direct physical link with $\lambda$. This is a great feature that can help in designing thin treatments. The resonators are flush mounted to the walls of the duct and thus act as “in parallel” elements. High ratios of $\lambda /l_A$ can be obtained using QWRs, especially for narrow ducts and if a single perfect absorption frequency is targeted. The resonators can even not be side-by-side which highlights that a “sub-wavelength long” FMAA is not necessarily acoustically compact. The only dimension of a FMAA composed of straight QWRs that is directly linked to $\lambda$ is its height which governs the resonance frequency of the resonators, i.e., for $\lambda \approx 4h$.

The term “broadband” indicates that a treatment is highly efficient over a relatively broad frequency range. The width of the high absorption frequency range of a FMAA is strongly related to the length of the FMAA and to the cross sectional area of the duct. Indeed, using a single pair of resonators, the smaller the section of the duct, the larger the absorption peak. Moreover, the combination of more than two resonators to enlarge the high absorption frequency range requires space and thus a longer FMAA. Thus, the frequency range of efficiency of a FMAA is important but does not speak for itself about the efficiency of the treatment in relation to the dimensions of the system.

The OAR is the ratio between the ventilated area, i.e., the cross sectional area of the duct, $S_d$, to the cross sectional area of the system composed of the duct and the FMAA. This parameter can be used to evaluate the ventilation performance of the FMAA in a qualitative manner. For a better accuracy, the pressure losses related to the morphology of the absorber should also be accounted for. In practice, the OAR is a convoluted way to differentiate FMAAs for ducts of very small cross sectional area from others. The OAR does not account for any acoustic parameter. A very large OAR can be obtained at the cost of high $l_A$ using QWRs folded along the axial direction of the duct.

This way, none of the mentioned terms appears satisfactory to describe the compactness of a FMAA, each of them should be interpreted with caution and should not diminish the importance of considering the raw dimensions of the system.

## Discussion

Passive FMAAs require at least two resonators to reach perfect upstream monochromatic absorption in the absence of duct geometry variation. By having a low resistance, the downstream resonator generates a soft wall around its resonance frequency that strongly reduces the transmission. The upstream resonator matches the impedance of the system with that of the air by having a certain impedance that depends on the target frequency, on the section of the duct, and on the spacing between the two resonators. The area of the duct section divided by the number of identical resonator at a given axial position and the spacing of the resonators govern their optimal resonance frequency and losses. Wide ducts lead to smaller frequency ranges efficiency for both resonators and require bulkier resonators than narrow ducts. Broadband absorption can be obtained by using multiple pairs of resonators, each pair being responsible for a given frequency. The resonators can be assembled in cascade or in parallel and, in this case, generate an equivalent resonator. In specific configurations, some resonators can cancel the reflection or the transmission at multiple frequencies. Also, the effects of the resonators away from their target frequency and the evanescent coupling between the resonators can strongly influence the FMAA behavior.

The evanescent coupling has a strong impact if the resonators are poorly damped or close to each other or if the cross section of the duct is large. This way, the design of compact and sub-wavelength FMAAs for broad frequency bandwidth in wide ducts is challenging. As demonstrated experimentally, the resonators that compose them should be relatively small and therefore quite damped to reduce the impact of evanescent coupling when using a compact absorber mounted in a wide duct. The resonators should also be arranged to maximize the distance between resonators of similar resonant frequency, i.e., they should not be arranged in a monotonic cascade of resonance frequency. Finally, the minimal length and height, along with the maximal OAR and frequency range of efficiency of a FMAA depend of the cross sectional area of the duct, the type of resonators forming the FMAA and the frequency range of interest.

## Methods

### Design of compact FMAA for wide duct

We focus on the design of compact FMAA and more particularly on the number of HRs. There are two reasons to replicate the resonators along the transverse direction of the waveguide instead of using a single large resonator per axial position. (i) The cavity volume of the HRs must not be too large to obtain relatively highly damped resonances and thus to maintain the evanescent coupling effects at a sustainable level. (ii) Even neglecting the evanescent coupling effects, the HRs must not be too large. We first consider a single HR of cavity width equal to that of the duct, $w_{c,y} = W$, of fixed neck and cavity heights, $h_n$ and $h_c$, and of optimized neck width, $w_n$, for maximal sound attenuation at a target frequency $f_0$. The larger the duct width, *W*, the lower the resulting TL peak. An increase of *W* leads to an increase of the HR cavity volume and thus to a decrease of its surface resistance at its resonance frequency, $Z(f_0)/w_n^2$. However, $Z(f_0)/w_n^2$ decreases slower than *H* increases. Then, as $|{T}(f_0)|$ is proportional to $HW\times Z(f_0)/w_n^2$, it increases with increasing *H*. This way, the HRs must usually form multiple rows to mimic a duct of relatively small width to maximize the TL of the FMAA.

Finally, the number of HRs columns (16) and HRs rows (8) have been selected after a performance parametric study of the considered system.

### The Transfer Matrix Method (TMM)

The TMM is a one dimensional lumped-element model considering that only plane waves propagate inside the duct and that there is no evanescent coupling between the resonators.

A transfer matrix $\mathbf{M}$ links the pressure *p* and axial flux $v_x$ at two axial positions along the duct^9^. For instance, between $x_1$ and $x_2>x_1$ we write

4 $$\begin{aligned} \begin{bmatrix} p \\ v_x \end{bmatrix}_{x_1} = \mathbf{M} \begin{bmatrix} p \\ v_x \end{bmatrix}_{x_2}. \end{aligned}$$

The transfer matrix of a rigid section of the duct of length $\delta$ and of cross-sectional area $S_d$ is

5 $$\begin{aligned} \mathbf{M} _\delta = \begin{bmatrix} \cos (k_0 \delta ) & \text {i}{\tilde{Z}}_0 \sin (k_0 \delta ) \\ \text {i}\sin (k_0 \delta )/{\tilde{Z}}_0 & \cos (k_0 \delta ) \end{bmatrix}, \end{aligned}$$

with ${\tilde{Z}}_0 = Z_0 / S_d$ and $Z_0$ the characteristic impedance of the air inside the duct. The following is the transfer matrix for the *i*-th point resonator with a surface impedance $Z_i$ (corrected by a length correction)^9^ and with a cross-sectional area connected to the duct $S_i$:

6 $$\begin{aligned} \mathbf{M} _i = \begin{bmatrix} 1 & 0 \\ 1/{\tilde{Z}}_i & 1 \end{bmatrix}, \end{aligned}$$

where ${\tilde{Z}}_i = Z_i / S_i$. If *G* resonators are located at the same axial location of the duct, i.e., assembled in parallel, they act as an equivalent resonator of impedance ${\tilde{Z}}_i$ such that

7 $$\begin{aligned} \frac{1}{{\tilde{Z}}_i} = \sum _{j=1}^G \frac{1}{{\tilde{Z}}_{i,j}}. \end{aligned}$$

A FMAA made of *F* (equivalent) resonators spaced by a distance $\delta _i$ is then described by a transfer matrix:

8 $$\begin{aligned} \mathbf{M} _{A}= \prod _{i=1}^F \mathbf{M} _{i} \mathbf{M} _{\delta _i}. \end{aligned}$$

Finally, its ${R}^+$, ${R}^-$ and *T* coefficients are then given by

9 $$\begin{aligned} {R}^+&= \frac{\mathbf{M }_{A}(1,1)-\mathbf{M }_{A}(2,2)+\mathbf{M }_{A}(1,2)/{\tilde{Z}}_0 - \mathbf{M }_{A}(2,1) {\tilde{Z}}_0}{\mathbf{M }_{A}(1,1)+\mathbf{M }_{A}(2,2)+\mathbf{M }_{A}(1,2)/{\tilde{Z}}_0 + \mathbf{M }_{A}(2,1) {\tilde{Z}}_0}, \end{aligned}$$

10 $$\begin{aligned} {R}^-&= \frac{-\mathbf{M }_{A}(1,1)+\mathbf{M }_{A}(2,2)+\mathbf{M }_{A}(1,2)/{\tilde{Z}}_0 - \mathbf{M }_{A}(2,1) {\tilde{Z}}_0}{\mathbf{M }_{A}(1,1)+\mathbf{M }_{A}(2,2)+\mathbf{M }_{A}(1,2)/{\tilde{Z}}_0 + \mathbf{M }_{A}(2,1) {\tilde{Z}}_0}, \end{aligned}$$

11 $$\begin{aligned} {T}&= \frac{2 \text {e}^{-\text {i}k_0 \sum _i \delta _i}}{\mathbf{M }_{A}(1,1)+\mathbf{M }_{A}(2,2)+\mathbf{M }_{A}(1,2)/{\tilde{Z}}_0 + \mathbf{M }_{A}(2,1) {\tilde{Z}}_0}. \end{aligned}$$

This way, the transfer matrix of the FMAA made of two resonators, HR$_1$ and HR$_2$, spaced by a distance $\delta$ is $\mathbf{M} _{A}= \mathbf{M} _{1} \mathbf{M} _\delta \mathbf{M} _{2}$. Its transmission and reflection coefficients are

12 $$\begin{aligned} {R}^+&= -\frac{\beta _1\text {e}^{-2\text {i}k_0\delta } + \beta _2 + \text {i}\sin (k_0\delta )\text {e}^{-\text {i}k_0\delta } }{2\beta _1 \beta _2 + \beta _1 + \beta _2 + \text {i}\sin (k_0\delta )\text {e}^{-\text {i}k_0\delta } }, \end{aligned}$$

13 $$\begin{aligned} {R}^-&= -\frac{\beta _1 + \beta _2\text {e}^{-2\text {i}k_0\delta } + \text {i}\sin (k_0\delta )\text {e}^{-\text {i}k_0\delta } }{2\beta _1 \beta _2 + \beta _1 + \beta _2 + \text {i}\sin (k_0\delta )\text {e}^{-\text {i}k_0\delta } }, \end{aligned}$$

14 $$\begin{aligned} {T}&= \frac{2\beta _1 \beta _2 \text {e}^{-\text {i}k_0\delta }}{2\beta _1 \beta _2 + \beta _1 + \beta _2 + \text {i}\sin (k_0\delta )\text {e}^{-\text {i}k_0\delta }}, \end{aligned}$$

with $\beta _1(f) ={\tilde{Z}}_1(f) /{\tilde{Z}}_0$ and $\beta _2(f)={\tilde{Z}}_2(f) /{\tilde{Z}}_0$. As the system is asymmetric, ${R}^+ \ne {R}^-$.

The expressions of optimal impedance of HR$_1$ and HR$_2$ for perfect absorption, Eqs. (1, 2, and 3), follow logically by considering that HR$_2$ cancels the transmitted wave and that, at a target frequency $f_0$, HR$_1$ cancels the reflected wave from the upstream side.

### The Mode Matching Technique (MMT)

A MMT *et al.* for cylindrical waveguides^27,28^ was adapted to rectangular section waveguides while still considering a modal basis composed of the rigid duct modes^17^. To enhance convergence rate of the method, the selected interpolation basis is formed by Chebychev polynomials of the first kind^29^ in this work.

The section of the waveguide is constant. The treatment is located on the $z=-H$ wall of the waveguide as depicted in Fig. 1. The resonators are replaced by their effective surface impedance and rigid walls correspond to an infinite surface impedance. No length correction at the opening of the resonators is required in the expression of the resonators impedance because the coupling is operated via the modes of the waveguide. The surface impedance is piecewise constant along the waveguide axial direction $\mathbf {x}$ and the transverse direction $\mathbf {y}$. The acoustic fields are projected onto the interpolation basis on each axial waveguide portion characterized by a specific wall impedance. Pressure and axial velocity are matched on the axial interfaces between waveguide sections.

The MMT equations used in this work are presented below. They are adapted from Refs.^17,28^, considering a rectangular waveguide and a Chebychev polynomial basis. Only the specificities of the interpolation basis are detailed hereafter.

In the *y* and *z* directions, the interpolating functions are

15 $$\psi _n(y) = \cos (n \arccos (2y/W+1)),~~n \in {\mathbb {N}} \ge 0, $$

16 $$\phi _m(z) = \cos (m \arccos (2z/H+1)),~~m \in {\mathbb {N}} \ge 0,$$

with $y \in [-W, 0]$ and $z \in [-H, 0]$. In practice, the convergence is ensured by considering the 15 first modes in each transverse direction of the waveguide. The pressure field *p*(*x*, *y*, *z*), the amplitude vector $\mathbf {P}$, and the interpolating functions are linked by

17 $$\begin{aligned} p(x,y,z) = \sum _{m,n} \phi _m(z) \psi _n(y) P_{mn}(x). \end{aligned}$$

In a portion of the waveguide, named $\Omega _{II}$, delimited by $x\in [l_1; l_2]$ and of surface impedance *Z*(*y*), the pressure modal differential equation is

18 $$\begin{aligned} \mathbf{M} \mathbf {P}''(x) + \mathbf{A} \mathbf {P}(x) = \mathbf {0}, \end{aligned}$$

with $\mathbf{M} ,\mathbf{A} , \mathbf{L}$, and $\mathbf{C}$ matrices equal to

19 $$\begin{aligned}&\mathbf{A} = \frac{\rho _0 \omega ^2}{K_0} \mathbf{M} - \mathbf{L} - {\text {i}\omega \rho _0} \mathbf{C} , \end{aligned}$$

20 $$\begin{aligned}&\mathbf{M} _{mn,m'n'} = \int _{-H}^0 \phi _m \phi _{m'} \text {d}z \int _{-W}^0 \psi _n \psi _{n'} \text {d}y, \end{aligned}$$

21 $$\begin{aligned}&\mathbf{L} _{mn,m'n'} = \int _{-H}^0\int _{-W}^0 \left( \frac{\text {d}\phi _m}{\text {d}z} \frac{\text {d}\phi _{m'}}{\text {d}z} \psi _n \psi _{n'} + \phi _m \phi _{m'} \frac{\text {d}\psi _n}{\text {d}y} \frac{\text {d}\psi _{n'}}{\text {d}y} \right) \text {d}z \text {d}y , \end{aligned}$$

22 $$\begin{aligned}&\mathbf{C} _{mn,m'n'} = \int _{-W}^0 \frac{1}{Z} \psi _n \psi _{n'} ~\text {d}y , \end{aligned}$$

with $\omega$ the angular velocity, $\rho _0$ and $K_0$ the density and bulk modulus of the air inside the waveguide, respectively. The solution of Eq. (18) takes the form^28^

23 $$\begin{aligned} \mathbf {P}(x) = \mathbf{X} \mathbf{D} (x-l_1) \mathbf {C}_1 + \mathbf{X} \mathbf{D} ^{-1}(x-l_2) \mathbf {C}_2, \end{aligned}$$

with $\mathbf{X}$ the matrix composed of the eigenvectors of $\mathbf{M} ^{-1}\mathbf{A}$ associated to the eigenvalues $d_n$, $\mathbf{D}$ a diagonal matrix with $\exp (-\text {i}\sqrt{d_n} x)$ on the diagonal, $\mathbf {C}_1$ and $\mathbf {C}_2$ amplitude vectors.

The domain $\Omega _{II}$ is surrounded by rigid portions of the waveguide, named $\Omega _{I}$ and $\Omega _{III}$. In these domains, the pressure modal differential equation is still given by Eq. (18) but $\mathbf{A}$ writes $\mathbf{A} _r = \frac{\rho _0 \omega ^2}{K_0} \mathbf{M} - \mathbf{L}$. Similarly, $\mathbf{X} _r$ is the matrix composed of the eigenvectors of $\mathbf{M} ^{-1}\mathbf{A} _r$ associated to the eigenvalues $c_n$.

The matrices $\mathbf{M}$ and $\mathbf{X} _r$ are not identity matrices because the considered interpolation basis is not orthonormal.

The pressure and axial velocity continuity conditions at the interfaces $\Omega _{I} - \Omega _{II}$ ($x=l_1$) and $\Omega _{II}- \Omega _{III}$ ($x=l_2$) are written as

24 $$\begin{aligned} \mathbf{X} _r (\mathbf {P}^+_I + \mathbf {P}^-_I)&= \mathbf{X} ( \mathbf {C}_1 + \mathbf{D} (\delta l) \mathbf {C}_2 ), \end{aligned}$$

25 $$\begin{aligned} \mathbf{X} _r (\mathbf {P}^+_{III} + \mathbf {P}^-_{III})&= \ \mathbf{X} ( \mathbf{D} (\delta l) \mathbf {C}_1 + \mathbf {C}_2 ), \end{aligned}$$

26 $$\begin{aligned} \mathbf{X} _r \mathbf{Y} _0 (\mathbf {P}^+_I - \mathbf {P}^-_I)&= \ \mathbf{X} \mathbf{Y} _{II} ( \mathbf {C}_1 - \mathbf{D} (\delta l) \mathbf {C}_2 ), \end{aligned}$$

27 $$\begin{aligned} \mathbf{X} _r \mathbf{Y} _0 (\mathbf {P}^+_{III} - \mathbf {P}^-_{III})&= \ \mathbf{X} \mathbf{Y} _{II} (\mathbf{D} (\delta l) \mathbf {C}_1 - \mathbf {C}_2 ), \end{aligned}$$

with $\delta l = l_2 - l_1$, $\mathbf{Y} _{0}$ and $\mathbf{Y} _{II}$ two diagonal matrices with $\sqrt{c_n}/{(\rho _0 \omega )}$ and $\sqrt{d_n}/{(\rho _0 \omega )}$ on the diagonal, respectively, and $\mathbf {P}_i^+$ (resp. $\mathbf {P}_i^-$) the modal amplitude vector of the forward (resp. backward) pressure wave in the domain $\Omega _i$ linked by the scattering matrix $\mathbf{S}$ such that

28 $$\begin{aligned} \begin{pmatrix} \mathbf {P}_{III}^+ \\ \mathbf {P}_{I}^- \end{pmatrix} = \mathbf{S} \begin{pmatrix} \mathbf {P}_{I}^+ \\ \mathbf {P}_{III}^- \end{pmatrix}. \end{aligned}$$

The scattering matrix is composed of $\mathbf{R} ^+$ and $\mathbf{T} ^+$ (resp. $\mathbf{R} ^-$ and $\mathbf{T} ^-$), the reflection and transmission modal matrices in the forward (resp. backward) direction, respectively:

29 $$\begin{aligned} \mathbf{S} = \begin{bmatrix} \mathbf{T} ^+ & \mathbf{R} ^- \\ \mathbf{R} ^+ & \mathbf{T} ^- \end{bmatrix}, \end{aligned}$$

with

30 $$\begin{aligned}&\mathbf{R} ^+ = \mathbf{R} ^- = [ \mathbf{G} - \mathbf{F} \mathbf{D} (\delta l) \mathbf{F} ^{-1}\mathbf{G} \mathbf{D} (\delta l) ]\cdot [ \mathbf{F} - \mathbf{G} \mathbf{D} (\delta l)\mathbf{F} ^{-1}\mathbf{G} \mathbf{D} (\delta l) ]^{-1}, \end{aligned}$$

31 $$\begin{aligned}&\mathbf{T} ^+ = \mathbf{T} ^- = [ \mathbf{F} \mathbf{D} (\delta l) - \mathbf{G} \mathbf{F} ^{-1}\mathbf{G} \mathbf{D} (\delta l) ]\cdot [ \mathbf{F} - \mathbf{G} \mathbf{D} (\delta l)\mathbf{F} ^{-1}\mathbf{G} \mathbf{D} (\delta l) ]^{-1} , \end{aligned}$$

and

32 $$\begin{aligned}&\mathbf{F} = \mathbf{X} _{r}^{-1} \mathbf{X} + (\mathbf{X} _{r}\mathbf{Y} _0)^{-1} \mathbf{X} \mathbf{Y} _{II}, \end{aligned}$$

33 $$\begin{aligned}&\mathbf{G} = \mathbf{X} _{r}^{-1}\mathbf{X} - (\mathbf{X} _{r}\mathbf{Y} _0)^{-1} \ \mathbf{X} \mathbf{Y} _{II}. \end{aligned}$$

In the rigid portions of the waveguide, the reflection and transmission matrices write

34 $$\begin{aligned} \mathbf{R} ^+&= \mathbf{R} ^- = \mathbf{0} , \end{aligned}$$

35 $$\begin{aligned} \mathbf{T} ^+&= \mathbf{T} ^- = \text {exp}_m(-\text {i}\sqrt{c_n} \delta l). \end{aligned}$$

### The Finite Element Model (FEM)

A finite element method (FEM) model of the waveguide lined by the FMAA is designed with COMSOL Multiphysics. The losses in the system only arise from the viscothermal losses of the HRs and are accounted for through equivalent fluids. The equation solved by using the FEM model is the generalized Helmholtz equation in fluids (air in the main waveguide or the equivalent fluids in the HRs),

36 $$\begin{aligned} \frac{\omega ^2}{K} p + \text {div}({{\rho }^{-1} \mathbf{grad} {p}}) = 0, \end{aligned}$$

where $\rho$ and *K* are the density and the bulk modulus of the fluid, respectively. In the domains corresponding to the neck and cavity of the HRs, $\rho$ and *K* are complex and frequency dependent. Their expressions are given by the Stinson’s model^30^.

### Optimization

Two optimization algorithms are used in this work: the heuristic Nelder-Mead (NM) algorithm^31^ implemented in the *Matlab* function *fminsearch* and the metaheuristic Particle Swarm Optimization (PSO) algorithm^32^ coded by Heris in *Matlab* language^33^. The PSO algorithm is an intelligent optimization algorithm well suited for optimization problems with large number of variables and possessing many local minima. The NM algorithm has a shorter execution time because it requires a lower number of evaluations of the cost function than the PSO algorithm. However, it is more likely to reach a local minimum especially if the number of variables of the problem is large and/or if it is initialized with a poor initial guess.

The optimizations of a single HR for TL$(f_0)=20$ dB or for maximal $\alpha ^+(f_0)$ are performed using the NM algorithm and the TMM predictive model. The designed FMAA for wide duct is obtained in two steps. First, the PSO algorithm and the MMT are used. An optimal set of parameters is found. Then, the NM algorithm is initialized with this set of parameters and is ran using the FEM model. In fact, the FEM model is more accurate than the MMT but requires a longer execution time to predict the behavior of the FMAA. Initializing the NM-FEM algorithm with the PSO-MMT results enhances the convergence of the optimization while guarantying a highly reliable numerical behavior prediction.

Table 2 summarizes the dimensions of the designed absorber. The values in italic correspond to parameters values that were fixed before the optimization to reduce the number of variables. They were chosen according to preliminary tests.

### Experimental set-up

The tested sample has been manufactured by means of the Stereolithography (SLA) 3D printing technique. It is made of a single epoxy part. The thickness of the walls is 0.8 mm.

The sample was tested in a in-house impedance tube^9^ of internal cross section $W \times H = 14.8 \times 15$ cm$^2$ and terminated by an anechoic termination. The sample is flush mounted to the tube inner wall. The reflection and transmission coefficients are derived from the diffusion matrix of the system. To reconstruct it, the pressure field inside the duct is measured twice: once with the sample oriented so that HR$_1$ is the closest resonator to the sound source (normal orientation) and once by reversing the orientation of the sample. The pressure field is measured at four upstream and four downstream positions by means of a unique moving microphone. The method accounts for the potential reflection induced by the anechoic termination of the duct.

### **Computation of**$Q_{leak}$

The quality factor $Q_{leak}$ due to the leakage of the resonators are computed by means of a complex frequency plane analysis of their behavior^34,35^. First, the reflection coefficient in the absence of losses of the studied flush mounted resonator is plotted in the complex frequency plane. Then, the complex frequency of the first pole of the resonator $\omega _{pole}$ is located. Finally, $Q_{leak}$ is computed as $Q_{leak}= 0.5 \text {Re}(\omega _{pole})/ \text {Im}(\omega _{pole})$. This way, $Q_{leak}$ depends on the properties of the resonator and on the dimensions of the duct.

## Data availability

The datasets used and/or analyzed during the current study available from the corresponding author on reasonable request.
